# Association of Preoperative Neutrophil-to-Lymphocyte Ratio and Immature Granulocytes with Postoperative Pain After Laparoscopic Sleeve Gastrectomy: A Retrospective Cohort Study

**DOI:** 10.3390/diagnostics16162579

**Published:** 2026-08-15

**Authors:** Mustafa Özdemir, Ahmet Aslan, Feyzanur Cavlak, Nurettin Yanık

**Affiliations:** 1 Department of Anesthesiology and Reanimation, Faculty of Medicine, Fırat University, 23119 Elazığ, Türkiye; 2 Department of General Surgery, Faculty of Medicine, Fırat University, 23119 Elazığ, Türkiye

**Keywords:** neutrophil-to-lymphocyte ratio, immature granulocytes, postoperative pain, bariatric surgery, sleeve gastrectomy, inflammation

## Abstract

**Background/Objectives**: Systemic inflammation may influence postoperative pain, but simple preoperative markers of pain risk are lacking in bariatric surgery. We examined whether the preoperative neutrophil-to-lymphocyte ratio (NLR) and immature granulocyte percentage (IG%) are associated with pain severity after laparoscopic sleeve gastrectomy (LSG). **Methods**: In this single-center retrospective cohort, records of 81 adults undergoing LSG were reviewed. Pain was rated on the 11-point numeric rating scale (NRS) in the recovery unit, at 24 h, and at 3 months. As NRS is ordinal, associations were assessed with Spearman correlation, covariate-adjusted ordinal logistic (proportional-odds) regression, NLR tertiles with a trend test, and receiver-operating-characteristic (ROC) analysis. **Results**: Preoperative NLR correlated positively with recovery unit NRS (rho = 0.36), 24 h NRS (rho = 0.37), and 3-month NRS (rho = 0.61; all *p* ≤ 0.001). After multivariable adjustment, log-NLR remained independently associated with recovery unit pain (odds ratio 6.1) and 24 h pain (odds ratio 8.1; both *p* < 0.001), with a graded relationship across tertiles (*p* for trend ≤ 0.020). For moderate-to-severe recovery unit pain, NLR discriminated with an area under the curve of 0.79 (optimal cutoff > 1.73). Neither IG% nor absolute IG was associated with any pain outcome (all *p* > 0.110). **Conclusions**: Higher preoperative NLR was independently and dose-dependently associated with greater early postoperative pain after LSG, whereas IG showed no association. NLR is a routinely available, essentially cost-free value that may help flag patients at risk of more severe early postoperative pain; prospective validation is warranted.

## 1. Introduction

Laparoscopic sleeve gastrectomy (LSG) is among the most frequently performed bariatric procedures worldwide, yet moderate-to-severe pain in the early postoperative period remains common despite minimally invasive access; in laparoscopic bariatric cohorts, the reported incidence of moderate-to-severe pain during the first 24 h reaches approximately 75% [1]. Inadequately controlled acute postoperative pain delays mobilization and recovery, increases opioid requirements, and is recognized among the risk factors for chronic postsurgical pain [2,3]. In a population with obesity—in whom opioid-related respiratory and other adverse effects are of particular concern—persistent postoperative opioid use after bariatric surgery is well documented [4]. Identifying, before surgery, the patients most likely to experience greater postoperative pain could therefore help individualize analgesic planning.

Surgical trauma initiates an inflammatory response, and locally released inflammatory mediators contribute to hyperalgesia and allodynia, linking systemic inflammation to postoperative pain perception [5]. The neutrophil-to-lymphocyte ratio (NLR), derived from a routine complete blood count, is an inexpensive and widely used marker of the systemic inflammatory state with established prognostic value across oncological, cardiovascular, and inflammatory conditions [5]. NLR has additionally been linked to pain and analgesic requirement in several surgical settings [5,6,7]. The immature granulocyte percentage (IG%), now reported automatically by modern hematology analyzers at no additional cost, reflects a left shift in granulopoiesis and has been proposed as an early indicator of acute inflammation and infection [8,9]. Because both indices are generated automatically by every routine preoperative complete blood count without additional sampling or cost, we evaluated them together, testing a priori whether either was associated with postoperative pain; the relationship of IG% with postoperative pain, in particular, has scarcely been studied.

Whether these readily available preoperative inflammatory markers are related to the severity of pain after LSG has not been clearly established. We therefore conducted a retrospective cohort study to examine the association of preoperative NLR and IG with postoperative pain severity—in the recovery unit, at 24 h, and at 3 months—in patients undergoing LSG. Because pain scores are ordinal, we analyzed them on their ordinal scale rather than dichotomizing them, to avoid loss of information and the use of arbitrary cut-points.

## 2. Materials and Methods

### 2.1. Study Design and Ethical Approval

This single-center retrospective cohort study was approved by the Non-Interventional Research Ethics Committee of Fırat University (approval no. 2026/02-51) and was conducted in accordance with the Declaration of Helsinki. All methods were performed in accordance with the relevant guidelines and regulations. The study is reported in accordance with the STROBE statement for observational studies [10,11]. Because of the retrospective design and the use of existing hospital records, the requirement for individual written informed consent was waived by the ethics committee, and institutional permission to access and use the de-identified records was granted. The records of consecutive adult patients who underwent elective LSG between November 2025 and January 2026 at our institution were reviewed.

### 2.2. Participants

Adults aged ≥18 years who underwent elective LSG for severe obesity were eligible. Consecutive patients with available preoperative complete blood count data, early postoperative pain assessments, and postoperative follow-up records were included. Exclusion criteria were conversion to open surgery, revisional bariatric surgery, emergency surgery, active infection at the time of surgery, known hematological disease, chronic inflammatory or autoimmune disease, malignancy, chronic systemic corticosteroid or immunosuppressive therapy, pregnancy, preoperative chronic opioid use, inability to reliably report pain scores, and missing data required for the primary analysis. Of 86 patients assessed for eligibility during the study period, 5 were excluded (3 for missing data, 1 for a rheumatologic/inflammatory disease, and 1 for revisional surgery), yielding a final sample of 81.

### 2.3. Anesthesia and Analgesia Protocol

All patients received a standardized general anesthetic. Anesthesia was induced with propofol, and intraoperative analgesia was provided by a continuous remifentanil infusion administered at a fixed rate; anesthesia was maintained with a volatile agent (desflurane or sevoflurane). No neuraxial block and no regional or local anesthetic technique (transversus abdominis plane block, surgical-site infiltration, or intraperitoneal local anesthetic) was used in any patient. Because the remifentanil infusion rate was fixed and essentially identical across patients (median ≈ 8.5 µg/min), the total intraoperative remifentanil dose was almost perfectly collinear with surgical duration (Pearson r = 1.00; median total dose 770 µg, interquartile range [IQR] 530–850). Postoperatively, no scheduled multimodal regimen and no routine non-steroidal anti-inflammatory drug were administered to all patients; rescue analgesia consisted of intravenous tramadol given on patient request when the patient reported pain (rather than triggered by a fixed NRS threshold), and no patient-controlled analgesia device was used. Total tramadol consumption during the first 24 h (mg) was recorded.

### 2.4. Data Collection and Variables

The following were extracted from the hospital records: demographic and clinical characteristics (age, sex, body mass index [BMI], American Society of Anesthesiologists [ASA] physical status, smoking, and alcohol use); preoperative laboratory values obtained from the most recent routine complete blood count before surgery, namely NLR (computed automatically as the absolute neutrophil/lymphocyte count ratio), IG%, and absolute IG count, measured on a Sysmex XN-1000 automated hematology analyzer (Sysmex Corporation, Kobe, Japan); intraoperative data (induction agent, intraoperative opioid and total dose, volatile agent, and surgical duration); and postoperative outcomes. Preoperative C-reactive protein and erythrocyte sedimentation rate were not part of the routine preoperative workup and were therefore not available for analysis.

### 2.5. Outcomes

The primary outcome was postoperative pain severity, assessed on the 11-point NRS (0–10) [12] at three time points: in the post-anesthesia care unit (recovery unit NRS), at 24 h, and at 3 months. Total analgesic consumption in the first 24 h was also recorded. For descriptive purposes, clinically meaningful moderate-to-severe pain was defined as NRS ≥ 4 [13]. The 3-month NRS was obtained from routine outpatient follow-up records and was analyzed as an exploratory secondary outcome. All patients were ASA III and all received intraoperative remifentanil; these variables therefore showed no between-patient variability.

### 2.6. Statistical Analysis

**Figure 1:**
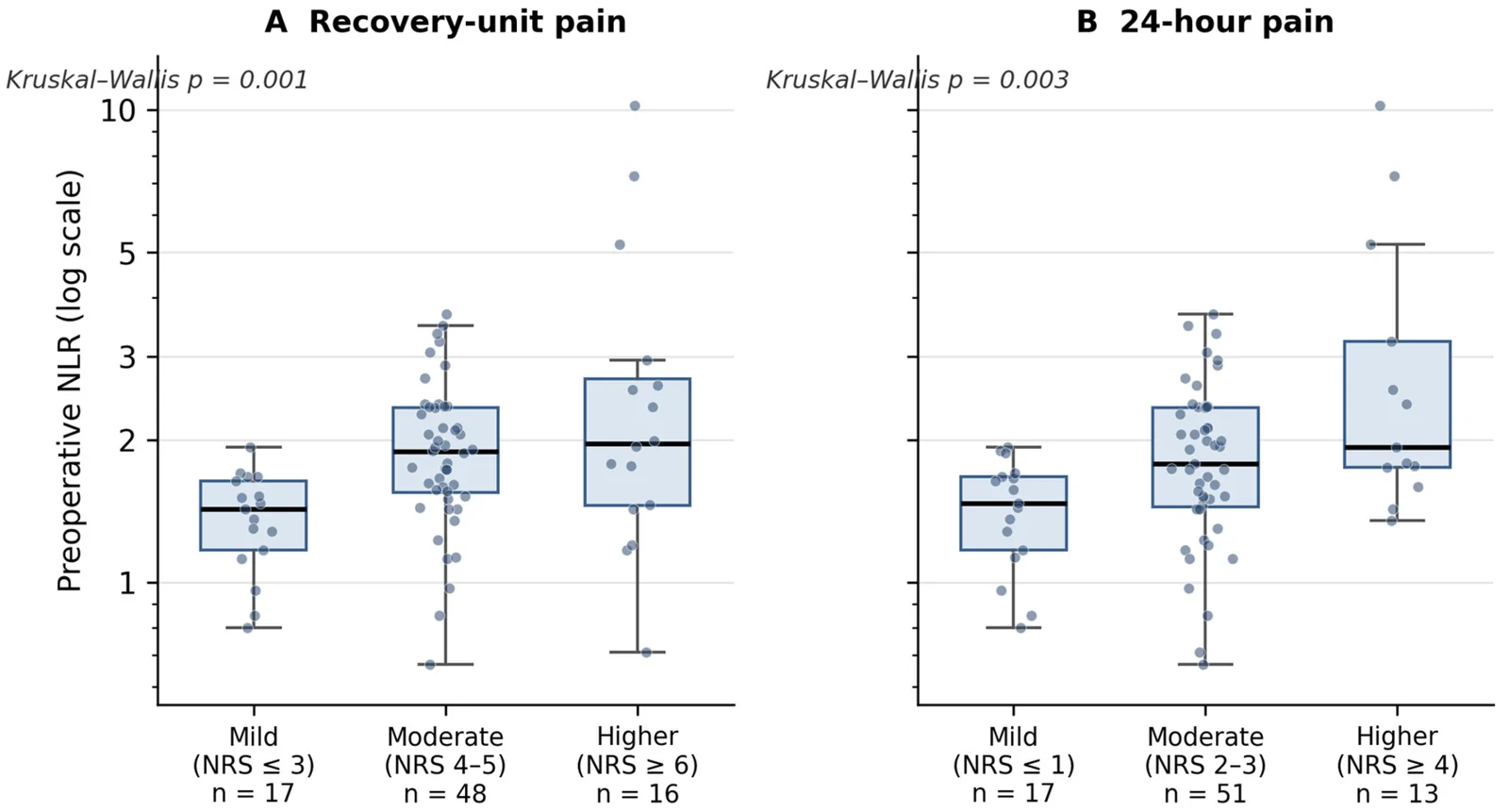
Preoperative neutrophil-to-lymphocyte ratio (NLR) across categories of postoperative pain severity. (**A**) Recovery unit pain (mild, numeric rating scale [NRS] ≤ 3, *n* = 17; moderate, NRS 4–5, *n* = 48; higher, NRS ≥ 6, *n* = 16). (**B**) 24 h pain (mild, NRS ≤ 1, *n* = 17; moderate, NRS 2–3, *n* = 51; higher, NRS ≥ 4, *n* = 13). Boxes show the median and interquartile range; whiskers extend to 1.5 times the interquartile range; points are individual patients (horizontally jittered). *p*-values are from the Kruskal–Wallis test. The y-axis is on a logarithmic scale.

Analyses were performed in Python 3.12 (SciPy 1.17, statsmodels 0.14, scikit-learn 1.5, and NumPy 2.4). The distribution of continuous variables was assessed with the Shapiro–Wilk test; because most variables (including NLR) deviated from normality, non-parametric and ordinal methods were used and continuous data are presented as median [IQR]. Categorical data are presented as number (percentage). Unadjusted associations between the markers (NLR, IG%, absolute IG) and each pain outcome were assessed with the Spearman rank correlation coefficient; for the 3-month NRS, which contained few distinct values and many ties, the Kendall tau-b coefficient was additionally reported as a tie-corrected estimate. The Benjamini–Hochberg procedure was applied to control the false-discovery rate across the correlation family [14]. Group differences in NLR across pain categories (Figure 1) were tested with the Kruskal–Wallis test.

For the recovery unit and 24 h NRS, covariate-adjusted ordinal logistic (proportional-odds) regression models [15] were fitted with log-transformed NLR as the primary exposure and the a priori covariates age, BMI, sex, smoking, surgical duration, and volatile agent. Because IG% violated the proportional-odds assumption (Brant test [16]), it was not retained in the primary adjusted model; instead, its association with pain was evaluated separately, in unadjusted analyses and as a sensitivity term added to the adjusted model. Total intraoperative remifentanil dose was not entered as a covariate because it was almost perfectly collinear with surgical duration (Pearson r = 1.00), reflecting the fixed-rate infusion described above. Because the 11-point scale contained sparse extreme categories, the proportional-odds assumption (Brant test [16]) and a sensitivity analysis of estimate stability were both evaluated on a clinically collapsed three-level outcome (mild, moderate, and higher pain). To assess a dose–response relationship without arbitrary dichotomization, NLR was additionally categorized into tertiles, and a test for trend was performed across tertiles; these tertile cut-points were derived empirically from the distribution of the present cohort (sample-specific) rather than from the established literature thresholds.

To evaluate the discriminative performance of preoperative NLR for clinically meaningful pain, receiver-operating-characteristic (ROC) curve analysis was performed for moderate-to-severe pain (NRS ≥ 4) at the recovery unit and 24 h time points. The area under the curve (AUC) was reported with a bootstrap 95% confidence interval (CI; 2000 resamples), and the optimal cut-off was identified by the Youden index with the corresponding sensitivity and specificity. Because of the small number of patients with clinically significant pain at 3 months (NRS ≥ 3, *n* = 2), the 3-month outcome was analyzed by correlation and descriptive statistics only; no regression or ROC analysis was performed for this outcome. A two-sided *p*-value < 0.05 was considered statistically significant.

During the preparation of this manuscript, a large language model (Claude; Anthropic, PBC) was used to generate Python code for the statistical analysis and to produce the figures; all code, analyses, and outputs were reviewed, executed, and verified by the authors, who take full responsibility for the results.

## 3. Results

### 3.1. Patient Characteristics

Eighty-one patients were included, with no missing data. Fifty-one (63.0%) were women, the median age was 40 years (IQR 28–50), and the median BMI was 42.0 kg/m^2^ (IQR 38.0–44.0); all patients were ASA physical status III. The median total intraoperative remifentanil dose was 770 µg (IQR 530–850). The preoperative NLR was right-skewed (median 1.73 [IQR 1.43–2.27]; range 0.67–10.20), and the IG% clustered at low values (median 0.30 [0.30–0.40]; range 0.10–1.90). Pain scores decreased over time (recovery unit NRS median 4 [4,5]; 24 h NRS 2 [2,3]; 3-month NRS 1 [0–2]). Using the prespecified threshold (NRS ≥ 4), 64 patients (79.0%) reported moderate-to-severe pain in the recovery unit and 13 (16.0%) at 24 h. On Shapiro–Wilk testing, all continuous variables except BMI deviated from normality (Table 1).

### 3.2. Unadjusted Association of NLR and IG with Pain

Preoperative NLR correlated positively and significantly with recovery unit NRS (rho = 0.356), 24 h NRS (rho = 0.372), and 3-month NRS (rho = 0.614; all *p* ≤ 0.001); all three associations remained significant after Benjamini–Hochberg correction. For the heavily tied 3-month outcome, the tie-corrected Kendall tau-b was 0.488 (*p* < 0.001), confirming the association. NLR was not significantly correlated with 24 h total analgesic (tramadol) consumption (rho = 0.185, *p* = 0.100). In contrast, neither IG% nor absolute IG was associated with any pain outcome (IG%: all rho ≤ 0.16, all *p* > 0.160; Table 2). Figure 1 displays preoperative NLR across categories of postoperative pain severity, with a significant difference across categories at both time points (Kruskal–Wallis *p* = 0.001 and *p* = 0.003, respectively).

### 3.3. Adjusted Analysis (Ordinal Logistic Regression)

Because the immature granulocyte percentage (IG%) violated the proportional-odds assumption (see below), the primary adjusted model comprised log-transformed NLR and the a priori covariates (age, BMI, sex, smoking, surgical duration, and volatile agent) without IG%. In this model, log-transformed NLR remained an independent correlate of a higher pain category for both recovery unit NRS (odds ratio [OR] 6.09, 95% CI 2.29–16.18; *p* < 0.001) and 24 h NRS (OR 8.08, 95% CI 3.00–21.81; *p* < 0.001), corresponding to an approximately fourfold increase in the odds of a higher pain category per doubling of NLR; among the covariates, only surgical duration (recovery unit model) reached significance (Table 3). The proportional-odds assumption was satisfied for this primary model (Brant test: omnibus *p* = 0.940 and *p* = 0.100 for the recovery unit and 24 h models, respectively; log-NLR *p* = 0.190 and *p* = 0.970). On a clinically collapsed three-level sensitivity outcome, the NLR effect remained large and significant (recovery OR 9.62, 95% CI 2.71–34.10; 24 h OR 11.17, 95% CI 3.29–37.91; both *p* < 0.001).

IG% was evaluated separately. It was not associated with pain in unadjusted analyses (Table 2), and when added to the adjusted model as a sensitivity check, it remained non-significant (recovery OR 0.46, *p* = 0.460; 24 h OR 2.22, *p* = 0.310) while being the only term to violate the proportional-odds assumption (Brant *p* = 0.001), consistent with its near-constant distribution; its inclusion left the NLR estimate essentially unchanged. IG% was therefore not retained in the primary model.

### 3.4. Dose–Response Across NLR Tertiles

When NLR was categorized into tertiles (T1 ≤ 1.52; T2 1.52–1.99; T3 > 1.99), the highest tertile was associated with significantly greater odds of higher pain than the lowest tertile for both recovery unit NRS (OR 3.41, 95% CI 1.20–9.67) and 24 h NRS (OR 4.17, 95% CI 1.53–11.36), with a significant trend across tertiles (*p* for trend = 0.020 and 0.005, respectively; Table 4).

### 3.5. Discriminative Performance of NLR (ROC Analysis)

**Figure 2:**
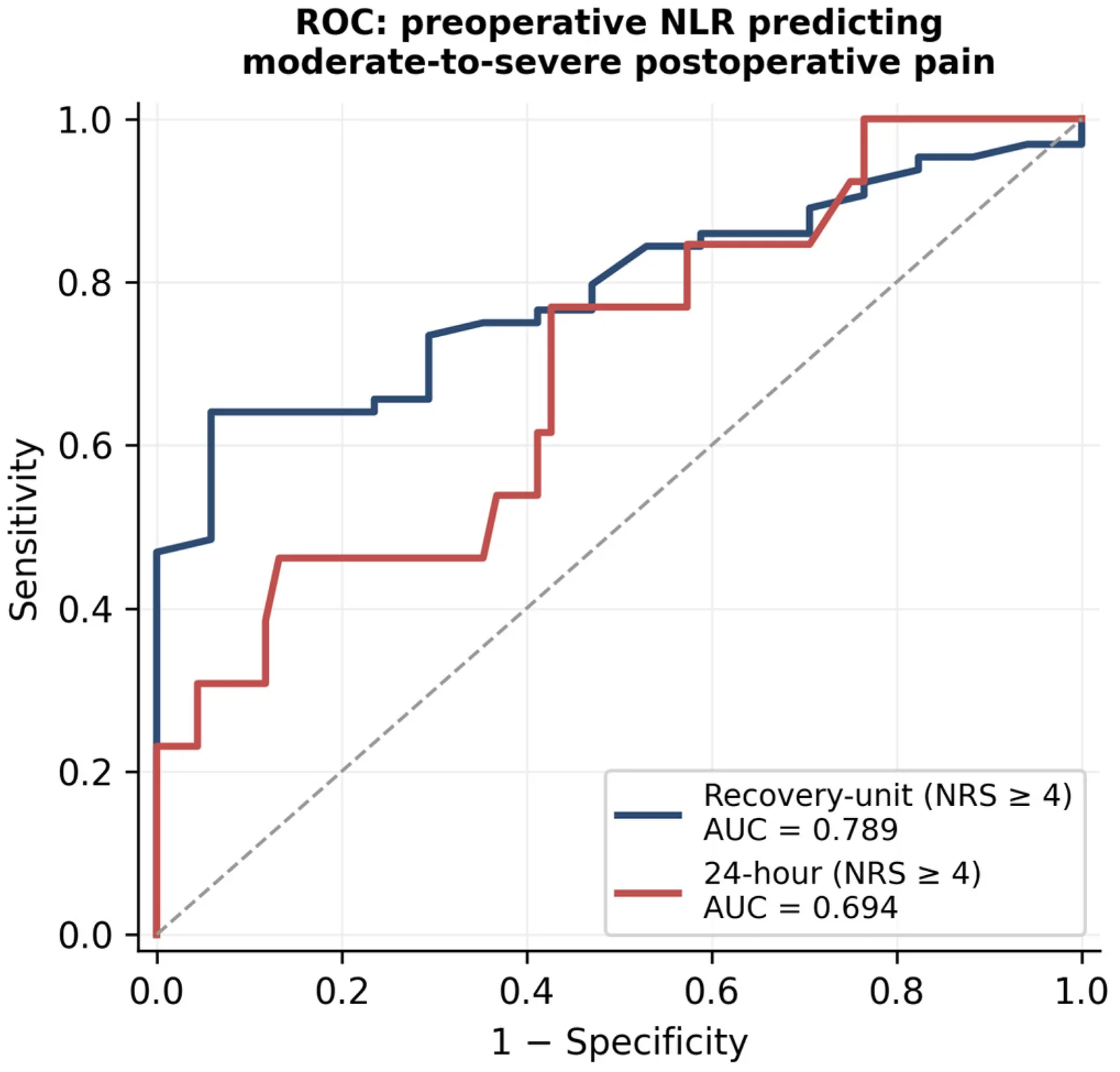
Receiver-operating-characteristic (ROC) curves for preoperative NLR predicting moderate-to-severe postoperative pain (NRS ≥ 4) in the Recovery unit and at 24 h. AUC, area under the curve. The diagonal reference line denotes no discrimination.

For moderate-to-severe pain (NRS ≥ 4), preoperative NLR discriminated with an AUC of 0.789 (95% CI 0.686–0.884) in the recovery unit and 0.694 (95% CI 0.526–0.852) at 24 h (Figure 2). The Youden-optimal cut-off was NLR > 1.73 for recovery unit pain (sensitivity 0.64, specificity 0.94) and NLR > 1.75 at 24 h (sensitivity 0.77, specificity 0.57) (Table 5). Notably, the identified cut-off (≈1.73) coincided with the cohort median, and these values should be regarded as exploratory and specific to this sample.

### 3.6. Three-Month Pain

At 3 months, most patients reported little or no pain (NRS 0, n = 27; NRS 1, n = 25; NRS 2, n = 27), and only two patients had an NRS ≥ 3. Median NLR increased across the 3-month NRS categories (1.51, 1.61, and 2.27 for NRS 0, 1, and 2, respectively). The strong NLR–3-month correlation was robust to exclusion of the two patients with NRS ≥ 3 (rho = 0.582). IG% was not associated with 3-month pain (rho = 0.154, *p* = 0.170).

## 4. Discussion

In this retrospective cohort of 81 patients undergoing LSG, a higher preoperative NLR was independently associated with greater postoperative pain severity, both in the recovery unit and at 24 h, and the association followed a graded, dose–response pattern across NLR tertiles. The relationship persisted after adjustment for age, BMI, sex, smoking, surgical duration, and volatile agent, remained stable in sensitivity analyses, and was reflected in a moderate discriminative performance on ROC analysis (recovery unit AUC 0.79). In contrast, neither the IG percentage nor the absolute IG count was associated with any pain outcome.

Our findings are consistent with a growing body of work linking this inexpensive inflammatory index to postoperative pain across diverse settings, including orthognathic surgery [5], cardiac surgery [6], joint arthroplasty [17], and degenerative spinal disease [7]; moreover, a higher perioperative NLR has been associated with chronic postsurgical pain after abdominal surgery [18], a setting close to ours. A plausible mechanism is that surgical trauma triggers a local and systemic inflammatory response in which mediators released by recruited and activated neutrophils sensitize nociceptors and contribute to hyperalgesia [5,19]; a higher preoperative NLR—reflecting relative neutrophilia and lymphopenia—may thus index a pro-inflammatory state that predisposes to more intense postoperative pain. The graded tertile relationship strengthens this interpretation, as a dose–response gradient is harder to attribute to chance than a single dichotomous contrast. Conversely, several studies have reported no such association: preoperative NLR was unrelated to postoperative pain after laparoscopic cholecystectomy [20] and did not predict pain or functional outcomes 12 months after lumbar fusion [21]. Differences in the surgical insult, the chronic low-grade inflammation characteristic of obesity [22], sample size, and analytic approach may account for these discrepancies.

Because intraoperative remifentanil can induce opioid-induced hyperalgesia and acute tolerance, its potential contribution to early postoperative pain warrants specific comment [23]. In this cohort, the remifentanil infusion was administered at a fixed rate that was essentially identical across patients, so the per-unit-time hyperalgesic exposure was uniform and the total dose varied only with surgical duration (Pearson r = 1.00), a covariate already included in the models. Remifentanil exposure therefore acted as a uniform background rather than a differential confounder of the NLR–pain association, and the high overall incidence of early moderate-to-severe pain (79% in the recovery unit) may in part reflect this shared hyperalgesic exposure. A corollary limitation is that, because no low-dose or remifentanil-free comparison group existed, the independent contribution of opioid-induced hyperalgesia to postoperative pain could not be quantified in this design.

We did not evaluate conventional inflammatory markers such as C-reactive protein or erythrocyte sedimentation rate, because these are not part of the routine preoperative workup for elective bariatric surgery at our center and were unavailable in this retrospective dataset. We deliberately focused on NLR and IG% precisely because they are generated automatically by every routine preoperative complete blood count at no additional cost or sampling—a practical advantage over markers that require dedicated testing. We do not claim that NLR is superior to conventional inflammatory panels; rather, we suggest that future prospective studies compare NLR head-to-head with C-reactive protein, erythrocyte sedimentation rate, and composite indices to establish its incremental predictive value.

The absence of any association between IG and postoperative pain is itself informative. IG was tested a priori because, like NLR, it is auto-reported on the complete blood count at no additional cost and reflects a granulocytic “left shift”; however, it rises most clearly with acute infection, sepsis, or marked systemic inflammation [8,9]. In our cohort of elective patients without active infection, IG values clustered at the low end of the measurement range (median 0.30%) with limited variability, leaving little dynamic range to detect a relationship with pain. Thus, IG likely does not capture the low-grade inflammatory variation relevant to nociception in this stable, non-infectious population, rather than indicating that inflammation is irrelevant to pain in general; transparent reporting of this negative result also guards against the selective emphasis on positive markers that can distort the literature.

While preoperative NLR was associated with NRS pain scores at recovery and 24 h, it was not significantly associated with 24 h total analgesic consumption. This divergence deserves emphasis and tempers any causal inference. In our setting, rescue tramadol was administered on patient request rather than being triggered by a standardized NRS threshold, and no patient-controlled analgesia device was used; consequently, recorded analgesic consumption reflected individual analgesic-seeking behavior, patient thresholds for requesting medication, and nursing workflow at least as much as underlying pain intensity. Analgesic consumption is therefore an indirect and heavily confounded surrogate that can track measured pain intensity poorly, and its dissociation from directly rated NRS is expected rather than contradictory.

The correlation between preoperative NLR and 3-month pain scores is intriguing but must not be over-interpreted. This outcome was included as an exploratory secondary endpoint, motivated by reports linking a higher perioperative NLR to chronic postsurgical pain [18]; however, only two patients reported clinically significant pain (NRS ≥ 3) at 3 months, so the finding reflects gradients among largely low residual pain scores rather than the prediction of chronic postsurgical pain, and no formal modeling was justified. Because the 3-month scores were drawn from routine outpatient records, they are additionally vulnerable to recall and documentation bias. Clinically, our data suggest that a routinely available, essentially cost-free preoperative value might help flag patients at risk of more severe early postoperative pain, who could then be prioritized for preemptive or intensified multimodal analgesia [2]. We emphasize, however, that these findings are hypothesis-generating: the wide confidence intervals, the single-center retrospective design, and the sample-derived, unvalidated NLR cut-off mean that NLR is not yet ready to guide individual clinical decisions.

An important consideration for interpretation is the analgesic context in which these associations were observed. Our institution did not employ a standardized multimodal analgesic protocol or regional/neuraxial techniques (e.g., transversus abdominis plane blocks), and rescue analgesia was limited to on-request tramadol; together with the fixed-rate remifentanil infusion and its potential for opioid-induced hyperalgesia, this likely contributed to the high incidence of moderate-to-severe early pain (79% in the recovery unit). Consequently, our findings should be regarded as specific to this setting, and it remains unknown whether the association between preoperative NLR and postoperative pain would persist under contemporary multimodal analgesia, in which lower and less variable pain scores might attenuate or mask the relationship. External validation in cohorts managed with standardized multimodal, opioid-sparing regimens is therefore needed before the findings can be generalized.

This study has several strengths and limitations. Strengths include analysis of pain on its native ordinal scale, adjustment for relevant confounders, demonstration of a dose–response relationship, false-discovery rate control, and transparent reporting of negative findings. Several limitations must also be acknowledged. First, the single-center, retrospective design with a modest sample (n = 81) limits generalizability, reduces the robustness of the multivariable models (yielding wide confidence intervals), and precludes causal inference. Second, postoperative analgesia was not delivered through a standardized scheduled multimodal protocol, and analgesic consumption was captured only as on-request tramadol, an imperfect surrogate. Third, all patients were ASA III and received a fixed-rate remifentanil infusion; while this homogeneity reduces confounding, it narrows applicability to other populations and anesthetic regimens, and the contribution of opioid-induced hyperalgesia could not be quantified. Fourth, conventional inflammatory markers (C-reactive protein, erythrocyte sedimentation rate) were unavailable, so the incremental value of NLR over standard markers could not be assessed. Fifth, the NLR tertile cut-points and the ROC-derived threshold were sample-specific and require external validation. Finally, the 3-month outcome was exploratory, based on very few patients with persistent pain, and drawn from routine records.

## 5. Conclusions

In this bariatric surgery cohort, a higher preoperative neutrophil-to-lymphocyte ratio was independently and dose-dependently associated with greater acute postoperative pain, whereas the immature granulocyte percentage—a marker seldom examined in relation to postoperative pain—showed no association, indicating that not all routinely available inflammatory indices carry the same information for pain. Because NLR is essentially cost-free and already present in every preoperative complete blood count, it is an attractive candidate for preoperative pain risk stratification in laparoscopic sleeve gastrectomy; however, given the single-center retrospective design, modest sample, and sample-derived thresholds, these exploratory findings require prospective, multicenter validation—ideally alongside conventional inflammatory markers and with predefined cut-offs—before clinical application.

## Figures and Tables

**Table 1:** Demographic and clinical characteristics (n = 81).

Variable	Value
Age (years), median [IQR]	40 [28–50]
Female sex, n (%)	51 (63.0)
BMI (kg/m^2^), median [IQR]	42.0 [38.0–44.0]
ASA III, n (%)	81 (100)
Smoking, n (%)	31 (38.3)
Alcohol use, n (%)	9 (11.1)
Surgical duration (min), median [IQR]	90 [60–100]
Total remifentanil dose (µg), median [IQR]	770 [530–850]
Volatile agent—desflurane/sevoflurane, n (%)	52 (64.2)/29 (35.8)
Preoperative NLR, median [IQR]	1.73 [1.43–2.27]
Preoperative IG (%), median [IQR]	0.30 [0.30–0.40]
Preoperative absolute IG (×10^9^/L), median [IQR]	0.03 [0.02–0.04]
Recovery unit NRS, median [IQR]	4 [4,5]
24 h NRS, median [IQR]	2 [2,3]
3-month NRS, median [IQR]	1 [0–2]
24 h tramadol consumption (mg), median [IQR]	100 [50–200]
Moderate-to-severe pain (NRS ≥ 4)—recovery/24 h, n (%)	64 (79.0)/13 (16.0)

ASA, American Society of Anesthesiologists; BMI, body mass index; IG, immature granulocyte; IQR, interquartile range; NLR, neutrophil-to-lymphocyte ratio; NRS, numeric rating scale. On Shapiro–Wilk testing, all continuous variables except BMI were non-normally distributed.

**Table 2:** Spearman correlations between preoperative markers and pain outcomes.

Marker	Recovery Unit NRS	24 h NRS	3-Month NRS
NLR	0.356 (*p* = 0.001)	0.372 (*p* < 0.001)	0.614 (*p* < 0.001)
IG (%)	0.134 (*p* = 0.230)	0.108 (*p* = 0.340)	0.154 (*p* = 0.170)

Values are Spearman rho (*p*). All three NLR associations remained significant after Benjamini–Hochberg correction. For the 3-month NRS, the tie-corrected Kendall tau-b was 0.488 (*p* < 0.001). NLR was not correlated with 24 h tramadol consumption (rho = 0.185, *p* = 0.100). Absolute IG was not associated with any outcome (all *p* > 0.110). IG, immature granulocyte; NLR, neutrophil-to-lymphocyte ratio; NRS, numeric rating scale.

**Table 3:** Covariate-adjusted ordinal logistic (proportional-odds) regression for postoperative pain (primary model).

Variable	Recovery Unit OR (95% CI)	*p*	24 h OR (95% CI)	*p*
log(NLR)	6.09 (2.29–16.18)	<0.001	8.08 (3.00–21.81)	<0.001
Age	0.99 (0.96–1.02)	0.500	0.99 (0.96–1.03)	0.692
BMI	0.95 (0.87–1.04)	0.248	1.11 (1.00–1.23)	0.054
Male sex	0.51 (0.21–1.26)	0.145	0.57 (0.23–1.40)	0.217
Smoking	2.27 (0.88–5.81)	0.089	1.95 (0.76–5.05)	0.167
Surgical duration	0.98 (0.96–1.00)	0.029	0.99 (0.97–1.01)	0.220
Desflurane (vs. sevoflurane)	1.38 (0.59–3.20)	0.456	1.19 (0.49–2.88)	0.701

NLR was entered as its natural logarithm. An odds ratio (OR) > 1 indicates higher odds of being in a higher pain category. The immature granulocyte percentage was not included in this primary model because it violated the proportional-odds assumption (Brant test); it was evaluated separately (see text). Total intraoperative remifentanil dose was excluded owing to near-perfect collinearity with surgical duration. BMI, body mass index; CI, confidence interval; NLR, neutrophil-to-lymphocyte ratio.

**Table 4:** Postoperative pain by NLR tertile (covariate-adjusted ordinal logistic regression).

NLR Tertile	Recovery Unit OR (95% CI)	24 h OR (95% CI)
T1 (≤1.52)	Reference	Reference
T2 (1.52–1.99)	1.32 (0.48–3.61)	1.62 (0.60–4.40)
T3 (>1.99)	3.41 (1.20–9.67)	4.17 (1.53–11.36)
*p* for trend	0.020	0.005

Adjusted for age, BMI, sex, smoking, surgical duration, and volatile agent. Tertile cut-points were sample-derived. BMI, body mass index; CI, confidence interval; NLR, neutrophil-to-lymphocyte ratio; OR, odds ratio.

**Table 5:** Discriminative performance of preoperative NLR for moderate-to-severe postoperative pain (NRS ≥ 4).

Time Point	AUC (95% CI)	Youden Cut-Off	Sensitivity	Specificity
Recovery unit	0.789 (0.686–0.884)	NLR > 1.73	0.64	0.94
24 h	0.694 (0.526–0.852)	NLR > 1.75	0.77	0.57

Cut-offs were identified by the Youden index and are sample-specific and exploratory. AUC, area under the receiver-operating-characteristic curve; CI, confidence interval (bootstrap, 2000 resamples); NLR, neutrophil-to-lymphocyte ratio; NRS, numeric rating scale.

## Data Availability

The data presented in this study are available on request from the corresponding author due to privacy restrictions concerning research participants.
